# Urine cell-based DNA methylation classifier for monitoring bladder cancer

**DOI:** 10.1186/s13148-018-0496-x

**Published:** 2018-05-30

**Authors:** Antoine G. van der Heijden, Lourdes Mengual, Mercedes Ingelmo-Torres, Juan J. Lozano, Cindy C. M. van Rijt-van de Westerlo, Montserrat Baixauli, Bogdan Geavlete, Cristian Moldoveanud, Cosmin Ene, Colin P. Dinney, Bogdan Czerniak, Jack A. Schalken, Lambertus A. L. M. Kiemeney, Maria J. Ribal, J. Alfred Witjes, Antonio Alcaraz

**Affiliations:** 1 Department of Urology Radboud University Medical Center, Nijmegen, The Netherlands; 2 Laboratory and Department of Urology, Hospital Clinic of Barcelona, IDIBAPS, University of Barcelona, Barcelona, Spain; 3 CIBERehd, Plataforma de Bioinformática, Centro de Investigación Biomédica en red de Enfermedades Hepáticas y Digestivas, Barcelona, Spain; 4 Saint John Emergency Clinical Hospital, Bucharest, Romania; 5 MD Anderson Cancer Center, Houston, Texas USA; 6 Hospital Clínic de Barcelona, Centre de Recerca Biomèdica CELLEX, office B22, C/Casanova, 143, 08036 Barcelona, Spain

**Keywords:** Cytology, Biomarkers, Bladder cancer, DNA methylation, Non-invasive diagnosis, Urine

## Abstract

**Background:**

Current standard methods used to detect and monitor bladder cancer (BC) are invasive or have low sensitivity. This study aimed to develop a urine methylation biomarker classifier for BC monitoring and validate this classifier in patients in follow-up for bladder cancer (PFBC).

**Methods:**

Voided urine samples (*N* = 725) from BC patients, controls, and PFBC were prospectively collected in four centers. Finally, 626 urine samples were available for analysis. DNA was extracted from the urinary cells and bisulfite modificated, and methylation status was analyzed using pyrosequencing. Cytology was available from a subset of patients (*N* = 399). In the discovery phase, seven selected genes from the literature (*CDH13*, *CFTR*, *NID2*, *SALL3*, *TMEFF2*, *TWIST1*, and *VIM2*) were studied in 111 BC and 57 control samples. This training set was used to develop a gene classifier by logistic regression and was validated in 458 PFBC samples (173 with recurrence).

**Results:**

A three-gene methylation classifier containing *CFTR*, *SALL3*, and *TWIST1* was developed in the training set (AUC 0.874). The classifier achieved an AUC of 0.741 in the validation series. Cytology results were available for 308 samples from the validation set. Cytology achieved AUC 0.696 whereas the classifier in this subset of patients reached an AUC 0.768. Combining the methylation classifier with cytology results achieved an AUC 0.86 in the validation set, with a sensitivity of 96%, a specificity of 40%, and a positive and negative predictive value of 56 and 92%, respectively.

**Conclusions:**

The combination of the three-gene methylation classifier and cytology results has high sensitivity and high negative predictive value in a real clinical scenario (PFBC). The proposed classifier is a useful test for predicting BC recurrence and decrease the number of cystoscopies in the follow-up of BC patients. If only patients with a positive combined classifier result would be cystoscopied, 36% of all cystoscopies can be prevented.

**Electronic supplementary material:**

The online version of this article (10.1186/s13148-018-0496-x) contains supplementary material, which is available to authorized users.

## Background

Seventy to 80% of patients with bladder cancer (BC) present with non-muscle-invasive tumors, either confined to the mucosa [stage Ta and carcinoma in situ (CIS)] or submucosa (stage T1). Based on clinical and pathological characteristics, non-muscle-invasive bladder cancer (NMIBC) patients can be classified into three different prognostic groups [1]. A minority of patients (20–30%) have low-risk tumors with a recurrence rate of 20–30%, without progression. The second and also the largest group, the intermediate-risk group, consists of patients who frequently develop a non-muscle-invasive recurrence (40–60%) but seldom progress to muscle-invasive disease. Finally, a small group of patients has a relatively aggressive NMIBC at presentation. The 5-year recurrence rate in this group is as high as 68% despite maximum intravesical treatment. Furthermore, up to 34% of these high-risk patients will develop muscle-invasive bladder cancer (MIBC) [2]. For this reason, an intensive follow-up schedule is mandatory in patients with intermediate- or high-risk NMIBC.

The follow-up schedule consists of urethrocystoscopy and urine cytology. Depending on the patient’s risk profile, the European Association of Urology guidelines recommend up to 15 urethrocystoscopies during the first 5 years of follow-up [3].

Urethrocystoscopy is considered the gold standard, but is invasive, expensive, and moreover misses up to 15% of the papillary and up to 30% of the flat recurrences [4, 5]. Urine cytology, on the other hand, has a high specificity (SP) but lacks sensitivity (SN) especially in low-risk tumors [6]. Additionally, the interobserver and intraobserver reproducibility of cytology is poor [7]. Recently, several non-invasive methods, NMP-22, bladder tumor antigen, and UroVysion FISH, have shown to help increase the sensitivity of urine cytology. However, due to limited specificity or sensitivity, the markers proposed to date have not been widely adopted in daily clinical practice. Therefore, there is a clear clinical need to find reliable markers to monitor the recurrence in NMIBC [8].

DNA methylation has been recognized to be important in developmental biology and cancer etiology in general [9]. DNA methylation occurs principally at CpG dinucleotides. These CpG dinucleotides are distributed throughout the genome, and the majority is normally methylated. Some regions in the genome have a high CpG density and are called CpG islands. Hypermethylation of normally unmethylated CpG islands in the promoter regions of tumor suppressor genes represses its transcription in human tumors [9, 10]. Therefore, aberrant DNA methylation is a potential biomarker for diagnosis, prognosis, and monitoring of disease after therapy [11]. Recently, it was shown that the combination of *SOX1*, *IRAK3*, and *L1-MET* provides better resolution than cytology and cystoscopy in the detection of early recurrence [12]. The objective of the present study is to investigate whether a set of methylation markers can lead to the development of a voided urine test that predicts tumor presence and may be used to stratify BC patients according to their risk of recurrence, thus allowing the reduction of the number of cystoscopies in the follow-up of BC.

## Methods

### Patients and clinical samples

After Institutional Review Board approval and obtaining patients’ informed consents, we prospectively collected freshly voided urine samples from BC patients, controls, and patients in follow-up for bladder cancer (PFBC) at four different centers [Hospital Clínic of Barcelona (Spain); Radboud University Medical Center in Nijmegen (The Netherlands); St. John Emergency Hospital, Bucharest (Romania); MD Anderson Cancer Center, Houston, Texas, (USA)], from October 2010 to February 2012. Participating centers were asked to collect and prepare the cell pellet by urine centrifugation and freeze them for a final processing at the Hospital Clinic of Barcelona or Radboud University Medical Center, Nijmegen. We took a two-stage approach with a discovery phase (or training set) and a validation phase (or testing set) (Additional file 1: Figure S1). In the discovery phase, the inclusion criteria for the cases were patients of both sexes, 18 years of age or older, and patients with histopathological confirmation of BC at any grade or stage. Without being mandatory, we recommended patients to have cytology at cystoscopy or during the period between cystoscopy and surgery. Patients with a prior endovesical chemotherapeutic or immunotherapeutic treatment could be included. The exclusion criteria were the absence of histological confirmation of BC and patients with other urological malignancies (prostate, kidney, urinary tract tumors). The inclusion criteria for the controls were patients of both sexes, 18 years of age or older, and with non-malignant urologic pathology (infection, lithiasis, urinary incontinence, BPH) or non-urologic pathology. The exclusion criterion for controls was a histological confirmation of any urological malignancy.

The validation phase was designed as a cross-sectional study including PFBC, i.e., the indicated population for the test in daily clinical practice. For efficiency reasons, we oversampled patients with a recurrence because we focused on sensitivity instead of specificity (see also the results in the “Reducing the number of follow-up cystoscopies by using the three-gene methylation/cytology combined classifier” section). PFBC with a prior endovesical chemotherapeutic or immunotherapeutic treatment could be included. Without being mandatory, we recommended PFBC to have cytology at cystoscopy. The exclusion criterion was a histological confirmation of any other urological malignancy.

A total number of 725 voided urine samples were prospectively collected by the four participating institutions. From the total number of urines collected, 99 (13%) were excluded from the study because of technical problems during the sample collection, storage, or analysis. Finally, 626 urines were used: 111 from BC patients and 57 from controls for the discovery phase and 458 from PFBC (of whom 173 had a recurrence) for the validation phase (Tables 1 and 2). The grade and stage of the tumors were determined according to WHO 2004 criteria and TNM 2002 classification, respectively [13, 14].

**Table 1:** Clinicopathological and demographic characteristics of the study population classified by the study phase

	Discovery phase	Validation phase
	Training set	Testing set
	*N* bladder cancer (%)	*N* R-PFBC (%)
Gender		
Male	86 (77)	135 (78)
Female	25 (23)	38 (22)
Age		
Mean	72	68
Range	39–98	26–99
Stage and grade		
Tis	7 (6)	13 (8)
Ta LG	26 (23)	61 (35)
Ta HG	11 (10)	20 12)
T1 LG	20 (18)	35 (20)
T1 HG	22 (20)	44 (25)
> T2 LG	1 (1)	–
> T2 HG	24 (22)	–
Subtotals	111	173
	N control (%)	
Gender		
Male	29 (51)	–
Female	28 (49)	–
Age		
Mean	60	–
Range	22–82	–
Urinary condition		
BPH	11 (19)	–
Urolithiasis	13 (23)	–
Incontinence	2 (4)	–
Benign bladder disease	1 (2)	–
Urinary tract infections	12 (21)	–
Non-urological diseases	18 (32)	–
Subtotals	57	–
		*N* NR-PFBC (%)
Gender		
Male	–	217 (76)
Female	–	68 (24)
Age		
Mean	–	69
Range	–	26–92
Stage and grade previous TURBT		
Tis	–	21 (7)
Ta LG	–	100 (35)
Ta HG	–	53 (19)
T1 LG	–	22 (8)
T1 HG	–	82 (29)
T2 HG	–	3 (1)
Tx LG	–	2 (1)
Tx HG	–	2 (1)
Subtotals	_–_	285
Total	168	458

*LG* low-grade, *HG* high-grade, *TURBT* transurethral resection bladder tumor, *BPH* benign prostate hyperplasia, *CIS/Tis* carcinoma in situ, *BC* bladder cancer, *R-PFBC* recurrent patients in follow-up for bladder cancer, *NR-PFBC* non-recurrent patients in follow-up for bladder cancer

**Table 2:** Clinicopathological and demographic characteristics of the study population classified by the participating center

		Hospital Clinic Barcelona	Radboud University Medical Center, Nijmegen	Saint John Emergency Clinical Hospital Bucharest	MD Anderson Cancer Center Houston	Total
Discovery phase	Bladder cancer urine samples					
	Stage					
	Tis	5	–	1	1	7
	Ta	14	5	5	13	37
	T1	19	2	21	–	42
	> T2	9	–	16	–	25
	Grade					
	LG	20	2	17	8	47
	HG	27	5	26	6	64
	Subtotal	47	7	43	14	111
	Control urine samples					
	BPH	8	3	–	–	11
	Urolithiasis	13	–	–	–	13
	Incontinence	1	1	–	–	2
	Benign bladder disease	4	5	1	–	10
	Urinary tract infection	1	8	4	–	13
	Non-urological diseases	6	–	–	2	8
	Subtotal	33	17	5	2	57
Validation phase	R-PFBC URINE SAMPLES					
	Stage					
	Tis	2	1	8	2	13
	Ta	13	15	47	6	81
	T1	4	2	55	18	79
	Grade					
	LG	14	8	74	–	96
	HG	5	10	36	26	77
	Subtotal	19	18	110	26	173
	NR-PFBC urine samples					
	Stage previous TURBT					
	Tis	5	6	6	4	21
	Ta	32	56	36	26	150
	Ta + CIS	1	–	–	2	3
	T1	36	16	25	19	96
	T1 + CIS	5	–	–	3	8
	T2	3	–	–	–	3
	Tx	4	–	–	–	4
	Grade previous TURBT					
	LG	34	35	38	19	126
	HG	52	43	29	35	159
	Subtotal	86	78	67	54	285
	TOTAL	185	120	225	96	626

*LG* low-grade, *HG* high-grade, *TURBT* transurethral resection bladder tumor, *BPH* benign prostate hyperplasia, *CIS/Tis* carcinoma in situ, *BC* bladder cancer, *R-PFBC* recurrent patients in follow-up for bladder cancer, *NR-PFBC* non-recurrent patients in follow-up for bladder cancer.

Tumors were classified according to their risk of recurrence and progression into three categories: high-risk (HR) NMIBC (any of the following: T1, HG/G3 tumors, or CIS), non-high-risk (nHR) NMIBC (all the other cases of NMIBC), and muscle-invasive bladder cancer (MIBC) (T2–4). None of the included patients had an upper urinary tract tumor.

### Urine sample processing

Urine samples were collected before cystoscopy, the day before the transurethral resection of the bladder tumor (TURBT), or the day before cystectomy. From all patients and controls, only one single sample was included.

#### For urine cytology

Urine cytology was performed according to Papanicolaou staining and evaluated by expert pathologists in each center blinded to the patient’s clinical history. The results were considered as positive or negative. No central cytology review was performed.

#### For methylation studies

Voided urine samples (50 to 100 ml) were collected in sterile containers containing 4 ml of 0.5 M EDTA, pH 8.0. Urines were immediately stored at 4 °C and processed within the next 24 h. The samples were centrifuged at 1000×*g* for 10 min, at 4 °C. The cell pellets were frozen at − 80 °C.

### DNA isolation, bisulfite treatment, and PCR

DNAs from the urinary cell pellets were extracted using QIAamp DNA Mini Kit (Qiagen) according to the manufacturer’s instructions and quantified with a NanoDrop1000 (NanoDrop Technologies, Wilmington, DE, USA). DNA extraction was performed in each center except for Bucharest, whose cell pellets were sent in dry ice to the Radboud University Medical Center, Nijmegen (The Netherlands) for DNA extraction.

One microgram of genomic DNA was used for the bisulfite modification using EpiTect Bisulfite kit (Qiagen, Inc.) following the manufacturer’s instructions. The modified DNA was eluted with 20 μl Tris-HCL (1 mM, pH 8.0) and stored at − 80 °C before further processing. Bisulfite modifications were performed in Hospital Clinic of Barcelona, Spain (training set), and in Radboud University Medical Center, Nijmegen, The Netherlands (testing set).

A total of seven DNA methylation markers, i.e., *CDH13*, *CFTR*, *NID2*, *SALL3*, *TMEFF2*, *TWIST1*, and *VIM2*, were selected from four recently published BC studies [15–18]. The sequences of the primers used to amplify the regions of interest of these genes and the PCR conditions are shown in Additional file 2: Table S1. PCR primers were designed using the PyroMark Assay Design software v2.0 (Qiagen). PCR was performed in a volume of 25 μl with 2 μl of converted genomic DNA, 0.6 U Ampli Taq Gold 360 DNA polymerase (Thermofisher), 0.8 μl of a mix of Primer-F and biotinylated Primer-R at 10 μM, 2 μl MgCl_2_ 25 mM, and 0.5 μl dNTPs 10 mM. Amplification was performed according to the following thermocycling conditions: denaturation at 95 °C for 10 min, followed by 45 cycles of 95 °C for 30 s, the optimal Tm for 30 s, and 72 °C for 1 min; and a final extension at 72 °C for 7 min. The formation of PCR products with accurate size was confirmed by resolving PCR samples (1 μl) by 2% agarose gel electrophoresis, with visualization by ethidium bromide staining.

### Pyrosequencing for quantitative methylation

Biotin-labeled single-stranded amplicons were isolated from 20 μl of the PCR product according to the protocol using the Pyromark Q96 Work Station and pyrosequenced with 0.3 μM sequence primer using PSQ96MD System (Biotage AB). Additional file 2: Table S1 shows the sequences of the primer sets used for bisulfite sequencing. The percent methylation for each of the CpGs was calculated using PyroQ CpG Software (Qiagen). The differences in the percentage of methylation were calculated between BC vs. control and recurrent vs. non-recurrent PFBC (R-PFBC and NR-PFBC, respectively) samples. A high correlation in the methylation percentages of the CpG dinucleotides in the same island was observed (Additional files 3 and 4: Figures S2 and S3). For this reason, hypermethylation was analyzed in all genes at the first CpG dinucleotide present (Additional file 5: Table S2).

### Data analysis

Univariable and multivariable logistic regression analyses were used to examine the associations between BC and DNA methylation status of urinary sediments. A forward stepwise logistic regression was performed to determine the best classifier between BC and control samples. The inclusion criterion was *p* value of < 0.1. Risk probability of presenting BC was calculated in the training set. We established cutoff point value (≥ 0.464) allowing 15% false negatives in the tumor group (SN = 85%). In the subset of samples in which cytology results were available, the cutoff point value that yielded 85% SN in the training set was 0.688, and in the combined model (methylation test + cytology results), it was 0.617. If the predicted probability value derived from each classifier in each of the samples was higher than the cutoff point value, the samples were classified for each of the classifiers as tumor sample. The performance of the models was evaluated in a testing set by means of AUROC using pROC R-package [19]. Student’s *t* test was used to evaluate statistical differences in DNA methylation. Statistical significance was established at *p* value of 0.05. R-software and SPSS v23.0 were used for calculations.

## Results

### Training set

**Fig. 1:**
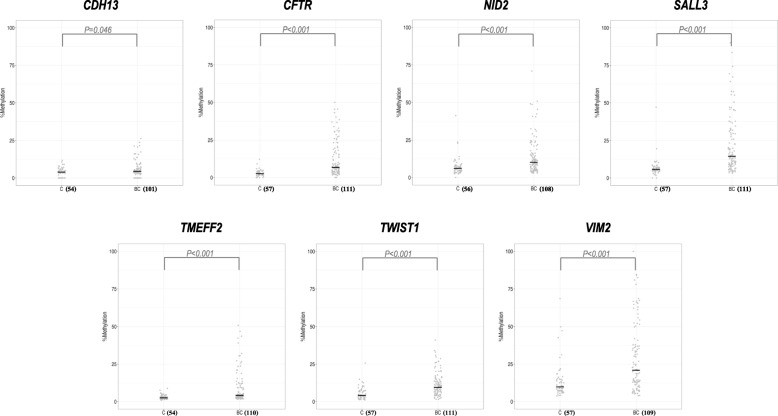
Percentage of DNA methylation in bladder cancer and control urine sediments for the seven selected genes analyzed in the discovery phase. The number of samples in each group is given in brackets. Abbreviations: BC, bladder cancer; C, control

**Fig. 2:**
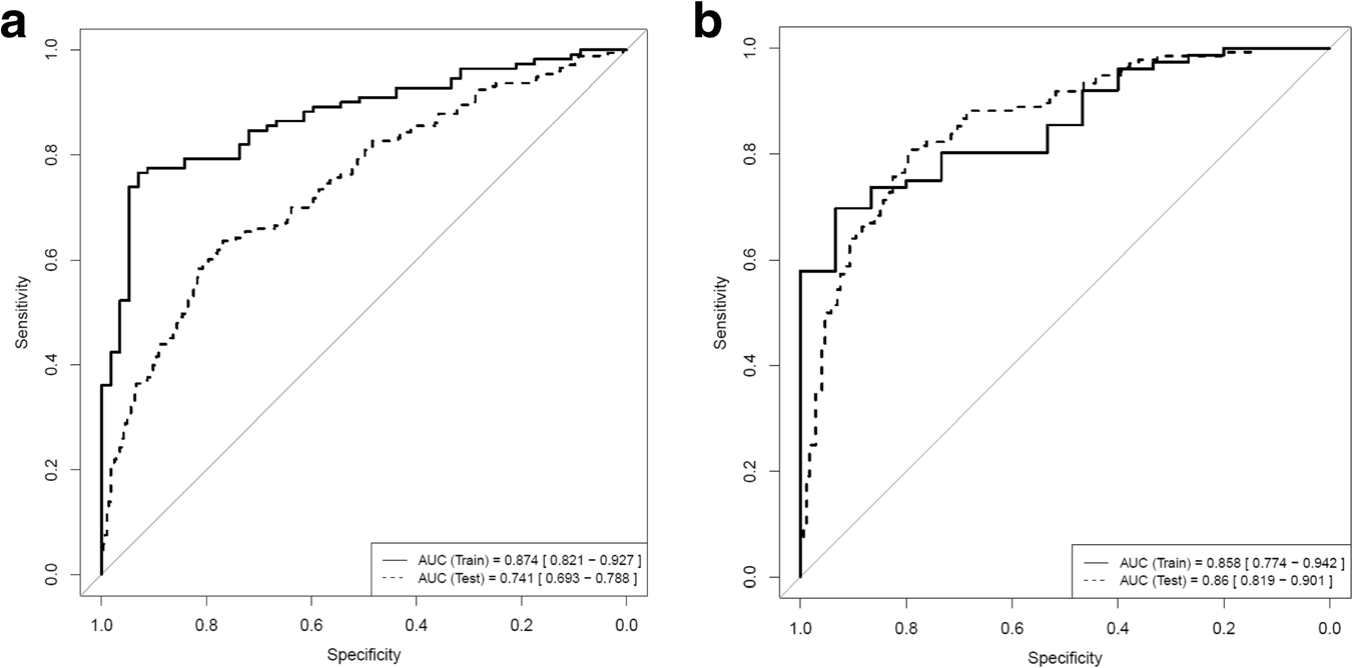
ROC curves in the training and testing series for (**a**) the three-gene BC methylation classifier and (**b**) the combined three-gene methylation/cytology classifier

DNA methylation of all seven selected genes was significantly increased in urine sediments from BC patients compared to controls (Fig. 1). To determine the combination of markers capable of detecting BC in urine sediments with the highest accuracy, we built a model of multiple markers by logistic regression. The best possible biomarker combination based on AUC was provided by the combination of *CFTR*, *SALL3*, and *TWIST1*. This three-gene methylation classifier achieved an AUC = 0.874 (Fig. 2a); at a fixed overall SN of 85%, the classifier provides a SP of 68%. Moreover, the SN of the three-gene methylation classifier increases through the BC risk groups and grading (Table 3).

**Table 3:** Diagnostic performance of the three-gene methylation classifier in the training and testing set of samples (at fixed sensitivity of 85% in the training set)

	Training set	Testing set
Overall		
*N* samples	111 BC/57 C	173 R-PFBC/285 NR-PFBC
AUC	0.874	0.741
SN (%)	84.68	89.6
SP (%)	68.42	30.53
PPV (%)	83.93	43.91
NPV (%)	69.64	82.86
Non-high-risk NMIBC		
*N* samples	26 BC/57 C	61 R-PFBC/285 NR-PFBC
SN (%)	73.08	88.52
SP (%)	68.42	30.53
PPV (%)	51.35	21.43
NPV (%)	84.78	92.55
High-risk NMIBC		
*N* samples	60 BC/57 C	112 R-PFBC/285 NR-PFBC
SN (%)	86.67	90.18
SP (%)	68.42	30.53
PPV (%)	74.29	33.78
NPV (%)	82.98	88.78
MIBC		
*N* samples	25 BC/57C	–
SN (%)	92	–
SP (%)	68.42	–
PPV (%)	56.1	–
NPV (%)	95.12	–
Low-grade		
*N* samples	47 BC/57 C	96 R-PFBC/285 NR-PFBC
SN (%)	76.6	90.62
SP (%)	68.42	30.53
PPV (%)	66.67	30.53
NPV (%)	78	90.62
High grade		
*N* samples	64 BC/57 C	77 R-PFBC/285 NR-PFBC
SN (%)	90.62	88.31
SP (%)	68.42	30.53
PPV (%)	76.32	25.56
NPV (%)	86.67	90.62

*LG* low-grade, *HG* high-grade, *AUC* area under the curve, *MIBC* muscle-invasive bladder cancer, *NMIBC* non-muscle invasive bladder cancer, *NPV* negative predictive value, *PPV* positive predictive value, *SN* sensitivity, *SP* specificity, *BC* bladder cancer, *C* control, *R-PFBC* recurrent patients in follow-up for bladder cancer, *NR-PFBC* non-recurrent patients in follow-up for bladder cancer.

### Testing set

**Fig. 3:**
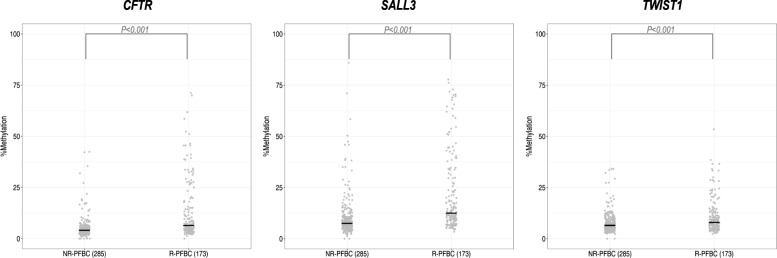
Percentage of DNA methylation in recurrent and non-recurrent PFBC urine sediments for the three genes of the classifier in the validation phase. The number of samples in each group is given in brackets. Abbreviations: NR-PFBC, non-recurrent patients in follow-up for bladder cancer; R-PFBC, recurrent patients in follow-up for bladder cancer

**Fig. 4:**
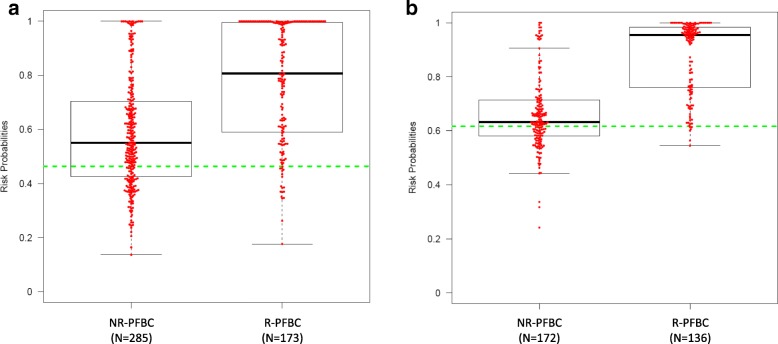
Box plots showing the individual risk probabilities derived from (**a**) the three-gene methylation classifier and (**b**) the combined three-gene methylation/cytology classifier for recurrent and non-recurrent PFBC in the cross-sectional study. Dots above the cutoff value (dashed line) denote positive samples, whereas those below signify negatives scores. Cutoff values for the three-gene methylation classifier and the combined three-gene methylation/cytology classifier are ≥ 0.464 and ≥ 0.617, respectively. Abbreviations: NR-PFBC, non-recurrent patients in follow-up for bladder cancer; R-PFBC, recurrent patients in follow-up for bladder cancer

To examine whether the three-gene methylation BC diagnostic classifier was able to identify recurrences in a clinical setting, the classifier was validated in an independent multicenter international series of 458 urine sediments from patients in follow-up for bladder cancer (PFBC), of whom 173 had a recurrence. Recurrent PFBC (R-PFBC; *N* = 173) displays higher percentages of DNA methylation compared with non-recurrent PFBC (NR-PFBC; *N* = 285) (Fig. 3). SN of the three-gene methylation BC classifier increased in the validation series (SN = 90%), while SP drops (SP = 31%), as evidenced by the ROC curves and AUC value (AUC = 0.741) (Table 3 and Fig. 2a). Figure 4a depicts the risk probabilities derived from the three-gene methylation classifier in R-PFBC and NR-PFBC.

### Comparison of test performance with urine cytology

A total of 399 urine cytologies (91 from the training and 308 from the testing set) were performed. In both training and testing set, SN of the three-gene methylation classifier (86 and 93%, respectively) was higher than that of the urine cytology (54 and 46%, respectively) in this subset of samples (Additional files 6 and 7: Table S3 and Figure S4). In the testing set, this means that 55% of the recurrences (68 out of 124) were detected by the three-gene classifier but were missed by urine cytology. On the other hand, 12 recurrences were missed by the three-gene classifier of which half was detected by cytology (Additional file 8: Figure S5). Negative predictive value (NPV) is also higher for the three-gene methylation classifier than that of the urine cytology in training (40 and 29%, respectively) and testing set (82 and 69%, respectively). Contrary, positive predictive value (PPV) is higher for the urine cytology than for the methylation classifier in training (98 and 89%, respectively) and testing set (85 and 50%, respectively). Cytology had a SP of 93 and 94% while the three-gene methylation classifier achieved a SP, in this subset of samples, of 47 and 27%, in the training and testing set, respectively. In the testing set, 120 NR-PFBC samples were positive by the three-gene methylation classifier. Nine of them also had positive urine cytology (Additional file 8: Figure S5). After 1 year, two NR-PFBC with a positive test (who had a negative cytology) had a tumor recurrence.

### Combination of the three-gene methylation classifier with urine cytology

Combining the three-gene methylation classifier and urine cytology results showed an improved diagnostic performance in the training (AUC 0.858) and as well as in the testing set (AUC 0.86) (Fig. 2b). A SN of 96% and a NPV of 92% are achieved in the testing set. Of note, in HG tumors, a 100% SN and NPV are achieved (Additional file 6: Table S3). The risk probabilities derived from the combined classifier in R-PFBC and NR-PFBC are shown in Fig. 4b.

### Reducing the number of follow-up cystoscopies by using the three-gene methylation/cytology combined classifier

In our study, we oversampled patients with BC. We therefore cannot directly calculate predictive values from the test results. In the hospitals participating in the study, recurrence is detected in approximately 10% of all follow-up cystoscopies performed (90% of patients previously diagnosed with NMIBC are without recurrence at the time of follow-up cystoscopy). In order to calculate the predictive values that reflect values in real clinical practice, we assumed the distribution of recurrent vs. non-recurrent to be 10 vs. 90%. For this, we multiplied the NR-PFBC samples by 7. Using the SN and SP that we found in the study, the PPV and NPV in the validation phase become 15 and 99%, respectively. If patients with a negative classifier will not undergo a cystoscopy, this means that more than a third (~ 36%) of all cystoscopies can be prevented at the cost of 4% of recurrences remaining undiagnosed which all were LG tumors.

## Discussion

In the present study, a set of DNA methylation markers to predict the presence of bladder cancer (BC) in urine samples has been selected. The best possible marker combination to discriminate BC from controls was the combination *CFTR*, *SALL3*, and *TWIST1*. We confirmed that these genes (and specifically CpG dinucleotides analyzed here) are hypermethylated in the bladder cancer tissue using methylation data from the TCGA Research Network [20] (Additional file 9: Figure S6). This supports their use as diagnostic markers in urine samples. In the training set, the three-gene methylation classifier achieved an AUC 0.874 while in the testing set, an AUC 0.741 was achieved to discriminate recurrent from non-recurrent patients in follow-up for bladder cancer (PFBC). These results improved significantly in the testing set when cytology results were included in the analysis (AUC 0.86).

*TWIST1* hypermethylation in BC was first described by Renard and co-workers [16]. In a case-control study (*n* = 145 cases/321 controls), they detected *TWIST1* and *NID2* hypermethylation in urine sediments of BC patients using methylation-specific PCR. This two-gene panel achieved a SN of 90%, SP of 93%, PPV of 86%, and NPV of 95%. Nevertheless, the group of Fantony published conflicting results. They found only a SN of 67% and SP of 69% for this two-gene urine panel [21]. However, the results were significantly better in the subgroup of active smokers. Unfortunately, we did not collect information about tobacco smoking, and therefore, we cannot perform a subset analysis for smoking behavior.

Yu and co-workers previously found in a case-control study that *CFTR* and *SALL3*, out of 59 genes that were screened, were the most frequently methylated genes to predict the presence of BC in urine [15]. However, in this study, no PFBC were included. In daily clinical practice, this group is especially of interest. It is not very likely that biomarkers will replace ureterocystoscopy in patients with macroscopic hematuria referred to the urologist. But in PFBC, urinary biomarkers with a high SN and high NPV could make a difference, i.e., lower the number of follow-up cystoscopies. Our combined three-gene methylation/cytology classifier achieves a high SN and NPV, for both high-risk and non-high-risk NMIBC. Consequently, more than a third of all cystoscopies could be prevented.

The SP of the combined classifier in the testing set, using PFBC samples, is expected to be lower than the SP of the training set, using BC and control samples. Possible reasons for methylation observed in PFBC samples are small tumors not yet detected by cystoscopy, residual tumor cells at the resection site, or epigenetically changed urothelial cells at the resection site or else in the bladder also known as epigenetic field defect [22]. In patients with persistent hypermethylation, which does not recur within 18 months, the presence of an epigenetic field defect is most likely. Wolff and co-workers suggested that the aberrant methylation is caused by a generalized epigenetic alteration in the whole bladder urothelium, and this widespread methylated urothelium may be the cause of the high recurrence rate in NMIBC [22].

The discovery of highly sensitive methylation markers allows us to lower the number of follow-up cystoscopies in more than a third of all follow-up patients. In the clinical situation, patients supply a urine sample before cystoscopy to perform the test. If the combined test is positive, patients will undergo a cystoscopy. In our validation series, 60% of NR-PFBC had a positive combined test and should undergo a cystoscopy. This is not a major problem since in normal daily practice, follow-up patients would have undergone a cystoscopy anyway. If the combined test is negative, cystoscopy could be skipped.

However, a methylation test has also financial and logistic implications, which means that a cost-effectiveness analysis is necessary. Using our three-gene methylation/cytology classifier, 4% of PFBC are wrongly diagnosed as not having a recurrence; all of them had LG NMIBC. Of note, cystoscopy, our gold standard, misses up to 15% of the papillary and up to 30% of the flat lesions [4].

The strengths of this study lie in the fact that we have chosen for a two-stage approach using PFBC in the validation phase. Furthermore, the use of voided urine samples to analyze the DNA methylation status allows the development of a non-invasive BC diagnostic tool with an easy translation into clinical practice. However, some limitations should be mentioned. To avoid inefficiency, patients with a recurrence were oversampled by also recruiting patients who were scheduled for a TURBT of a proven bladder tumor. Consequently, the number of NR-PFBC was misrepresented in the validation series, and we had to make an estimation to calculate the number of cystoscopies that could be skipped. Secondly, 13% of the samples had to be excluded due to technical failures. Thirdly, intravesical treatments in NMIBC patients may have influenced methylation patterns. Finally, we have evaluated only a limited number of hypermethylated genes with diagnostic value previously described in the literature.

## Conclusions

In conclusion, this combined three-gene methylation/cytology classifier can reduce the number of follow-up cystoscopies in PFBC. This approach may improve the patients’ quality of life. For a definitive conclusion, replication of the classifier in another series of patients and cost-effectiveness studies are needed.

## Additional files

Additional file 1: **Figure S1.** Flowchart of the entire study. A total of seven hypermethylated genes, differentially expressed between BC patients and controls (*n* = 168), were determined in the discovery phase. With these results, a three-gene methylation classifier was developed. This three-gene classifier was tested in a cross-sectional study (validation phase; *n* = 458). Samples with available cytology results in each phase are indicated. Abbreviations: BC, bladder cancer; C, control; R-PFBC, recurrent patients in follow-up for bladder cancer; NR-PFBC, non-recurrent patients in follow-up for bladder cancer. (PPTX 75 kb)

Additional file 2: **Table S1.** Primer sequences used in PCR and pyrosequencing. (DOCX 13 kb)

Additional file 3: **Figure S2.** Pearson correlation coefficient heat map of the percentage of methylation for every CpG dinucleotide in the seven genes. Every CpG sites are correlated (via the Pearson correlation) with all others. Correlations are scaled by the color of the corresponding cell. Parameters are represented in the same order on the x- and y-axes. (PPTX 1232 kb)

Additional file 4: **Figure S3.** Representative pyrograms showing gene methylation patterns in DNA urine samples from a bladder cancer patient. Percentage of methylation is indicated above peaks (gray columns) corresponding to the CpG sites in this region. (PPTX 183 kb)

Additional file 5: **Table S2.** Percentage of methylation for each CpG dinucleotide in the seven selected genes in control and bladder cancer urine samples. Underlined in grey the CpG site used for methylation analysis. Abbreviations: SDV; Standard Deviation. (DOCX 21 kb)

Additional file 6: **Table S3.** Diagnostic performance of the three-gene methylation classifier, cytology, and the combined methylation/cytology classifier in the training and testing subset of samples with cytology available. Abbreviations: LG, Low Grade; HG, High Grade; AUC, area under curve; MIBC, muscle invasive bladder cancer; NMIBC, Non-Muscle Invasive Bladder Cancer; NPV, Negative Predictive Value; PPV, Positive Predictive Value; SN, Sensitivity; SP, Specificity; BC, Bladder Cancer; C, Control; R-PFBC, Recurrent Patients in Follow up for Bladder Cancer; NR-PFBC, Non Recurrent Patients in Follow up for Bladder Cancer. (DOCX 20 kb)

Additional file 7: **Figure S4.** Sensitivity, negative and positive predictive values of urine cytology, the three-gene methylation classifier, and the combined three-gene methylation/cytology classifier in the testing set (*N* = 308). Overall specificity was 94% for urine cytology, 27% for the three-gene methylation classifier, and 40% for the combined three-gene methylation/cytology classifier. Abbreviations: LG, low-grade; HG, high-grade; NMIBC nHR, non-muscle-invasive bladder cancer non-high risk; NMIBC HR, non-muscle-invasive bladder cancer high risk; NPV, negative predictive value; PPV, positive predictive value. (PPTX 189 kb)

Additional file 8: **Figure S5.** Flow diagram of participants in the cross-sectional study according a) to the three-gene methylation classifier and cytology results and b) to the combined three-gene methylation/cytology classifier. Abbreviations: R-PFBC, recurrent patients in follow-up for bladder cancer; NR-PFBC, non-recurrent patients in follow-up for bladder cancer; Cytol, cytology; NA, non-available; Test, combined three-gene methylation/cytology classifier. (PPTX 85 kb)

Additional file 9: **Figure S6.** DNA methylation profiles for bladder cancer and control tissue samples for the three-gene classifier. Data obtained from Wanderer Web page: http://maplab.imppc.org/wanderer/. The red arrow indicates the CpG dinucleotide analyzed in each of the three genes. (PPTX 203 kb)

## Abbreviations

AUC: Area under the curve
BC: Bladder cancer
BPH: Benign prostatic hyperplasia
C: Control
CIS/Tis: Carcinoma in situ
HG: High-grade
HR: High-risk
LG: Low-grade
MIBC: Muscle-invasive bladder cancer
NA: Not available
nHR: Non-high risk
NMIBC HR: Non-muscle-invasive bladder cancer high risk
NMIBC nHR: Non-muscle-invasive bladder cancer non-high risk
NMIBC: Non-muscle-invasive bladder cancer
NPV: Negative predictive value
NR-PFBC: Non-recurrent patients in follow-up for bladder cancer
PPV: Positive predictive value
R-PFBC: Recurrent patients in follow-up for bladder cancer
SN: Sensitivity
SP: Specificity
TURBT: Transurethral resection of the bladder tumor
