# Supplementary figures and images for: Urine cell-based DNA methylation classifier for monitoring bladder cancer
Source: Clin Epigenetics. 2018 May 30;10:71. doi: 10.1186/s13148-018-0496-x (PMC5975622; doi:10.1186/s13148-018-0496-x)

## Slide 1
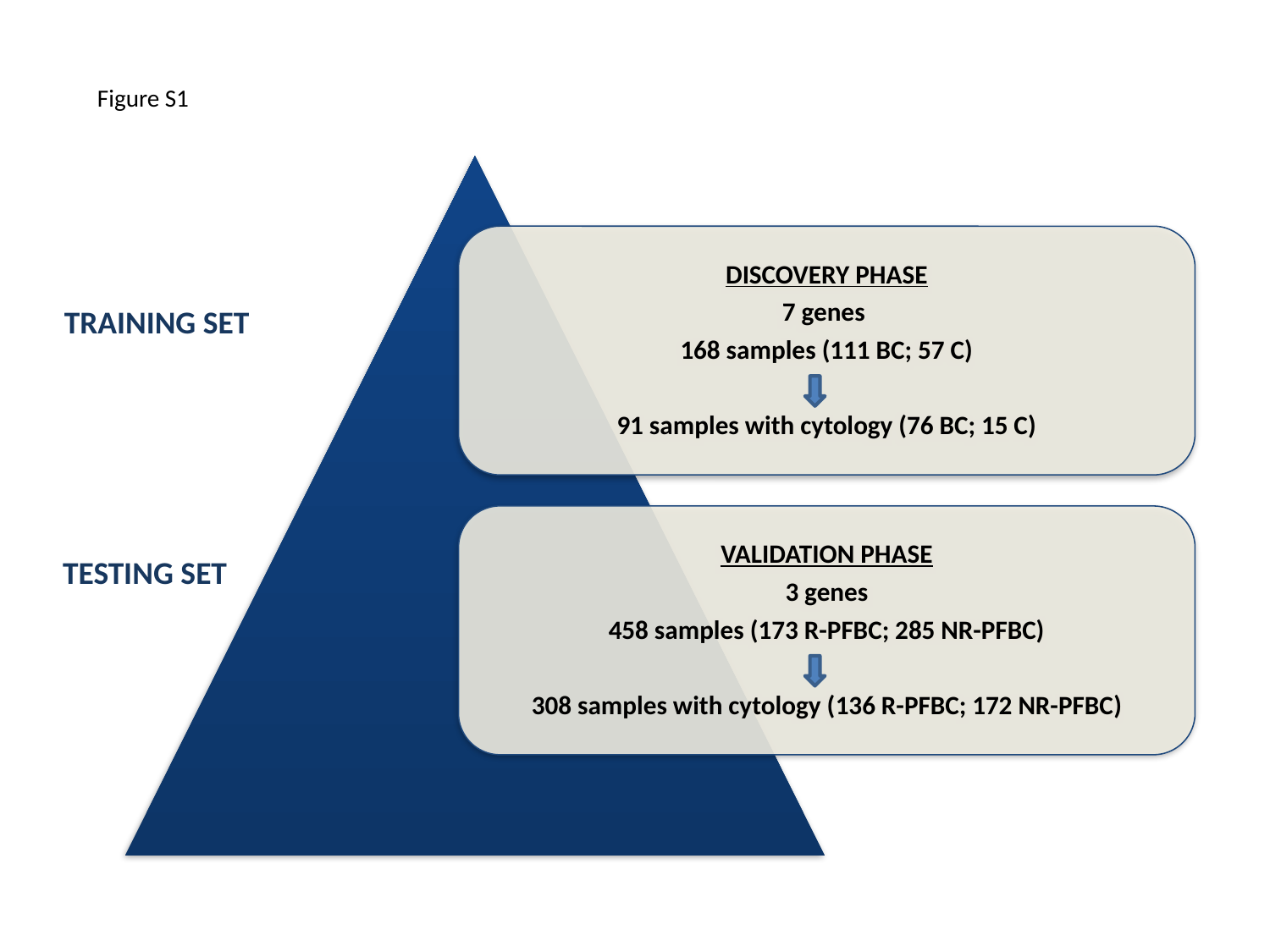

Figure S1
TRAINING SET
TESTING SET

Supplement: Supplementary file 1 — Figure S1. Flowchart of the entire study. A total of seven hypermethylated genes, differentially expressed between BC patients and controls (n = 168), were determined in the discovery phase. With these results, a three-gene methylation classifier was developed. This three-gene classifier was tested in a cross-sectional study (validation phase; n = 458). Samples with available cytology results in each phase are indicated. Abbreviations: BC, bladder cancer; C, control; R-PFBC, recurrent patients in follow-up for bladder cancer; NR-PFBC, non-recurrent patients in follow-up for bladder cancer. (PPTX 75 kb) [file 13148_2018_496_MOESM1_ESM.pptx]

## Slide 1
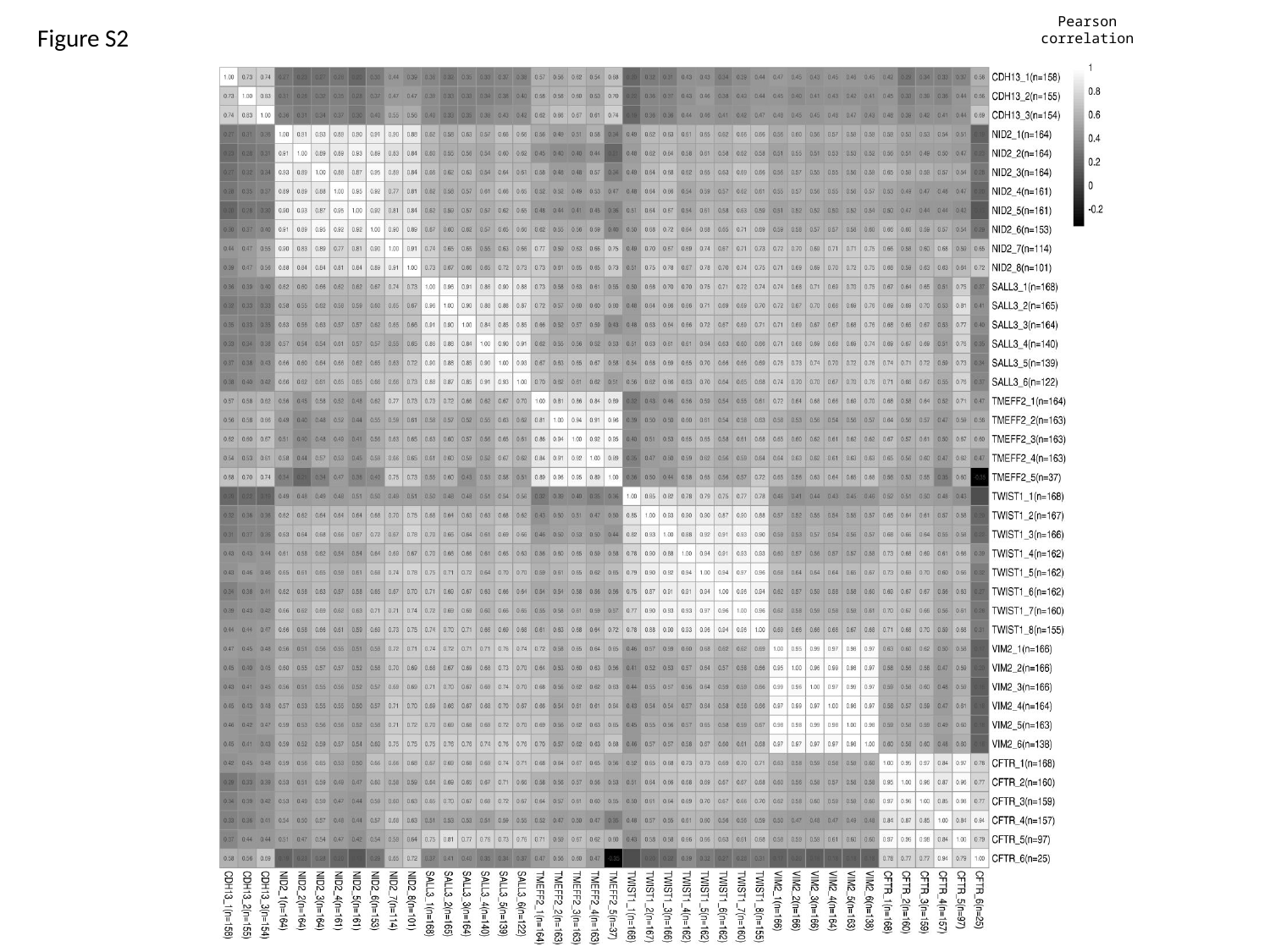

Pearson correlation
Figure S2

Supplement: Supplementary file 3 — Figure S2. Pearson correlation coefficient heat map of the percentage of methylation for every CpG dinucleotide in the seven genes. Every CpG sites are correlated (via the Pearson correlation) with all others. Correlations are scaled by the color of the corresponding cell. Parameters are represented in the same order on the x- and y-axes. (PPTX 1232 kb) [file 13148_2018_496_MOESM3_ESM.pptx]

## Slide 1
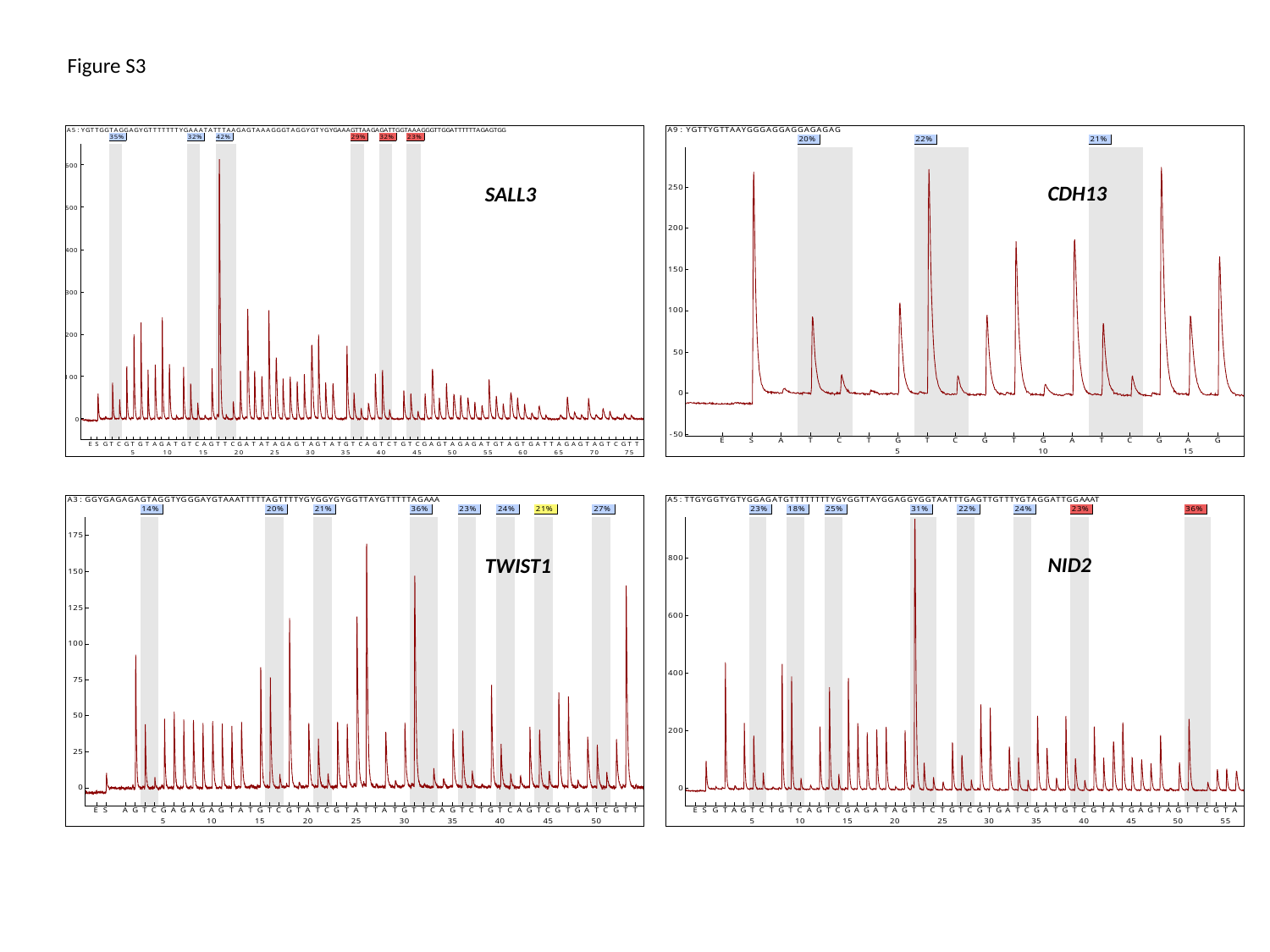

Figure S3
CDH13
SALL3
NID2
TWIST1

## Slide 2
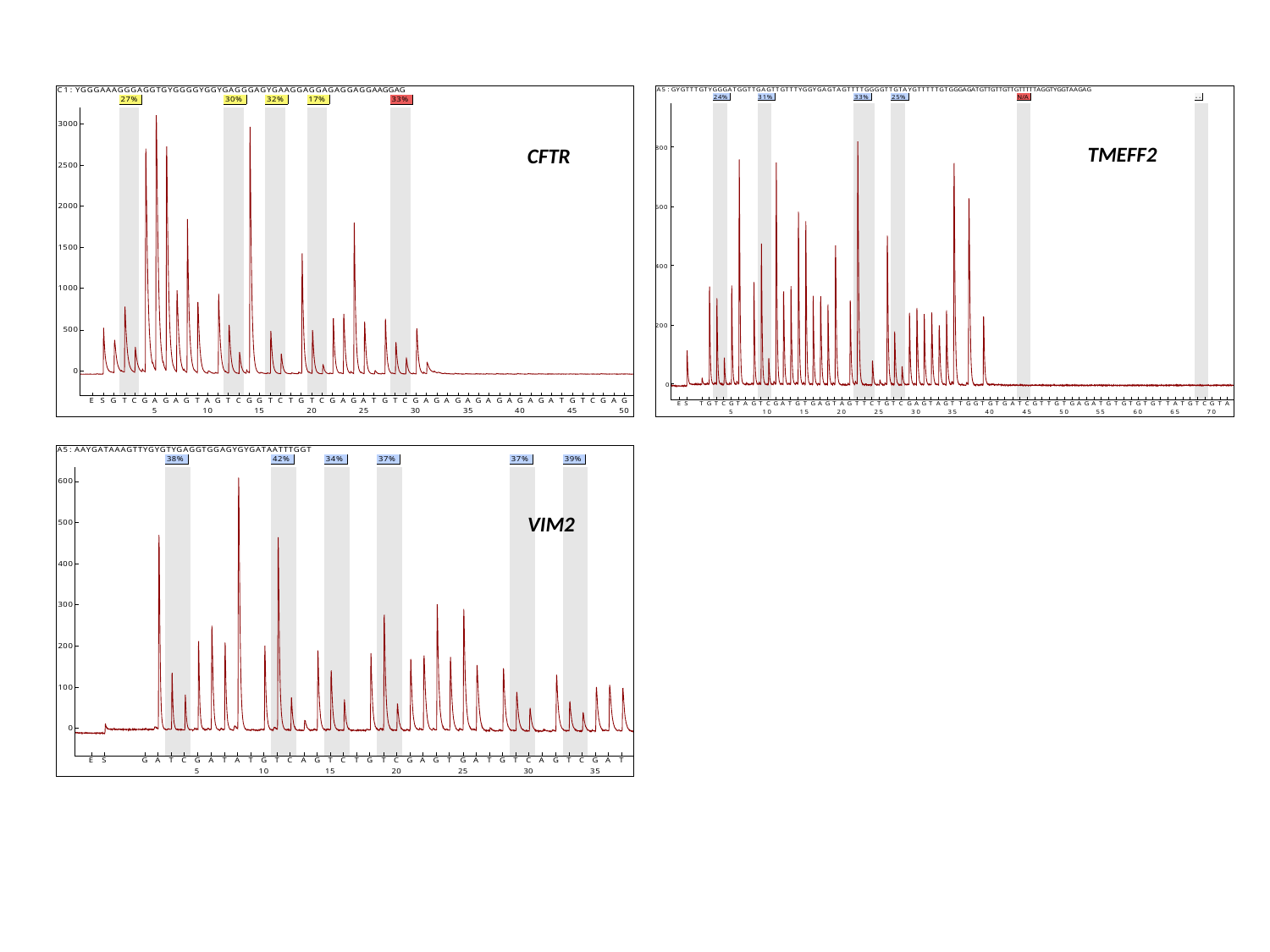

TMEFF2
CFTR
VIM2

Supplement: Supplementary file 4 — Figure S3. Representative pyrograms showing gene methylation patterns in DNA urine samples from a bladder cancer patient. Percentage of methylation is indicated above peaks (gray columns) corresponding to the CpG sites in this region. (PPTX 183 kb) [file 13148_2018_496_MOESM4_ESM.pptx]

## Slide 1
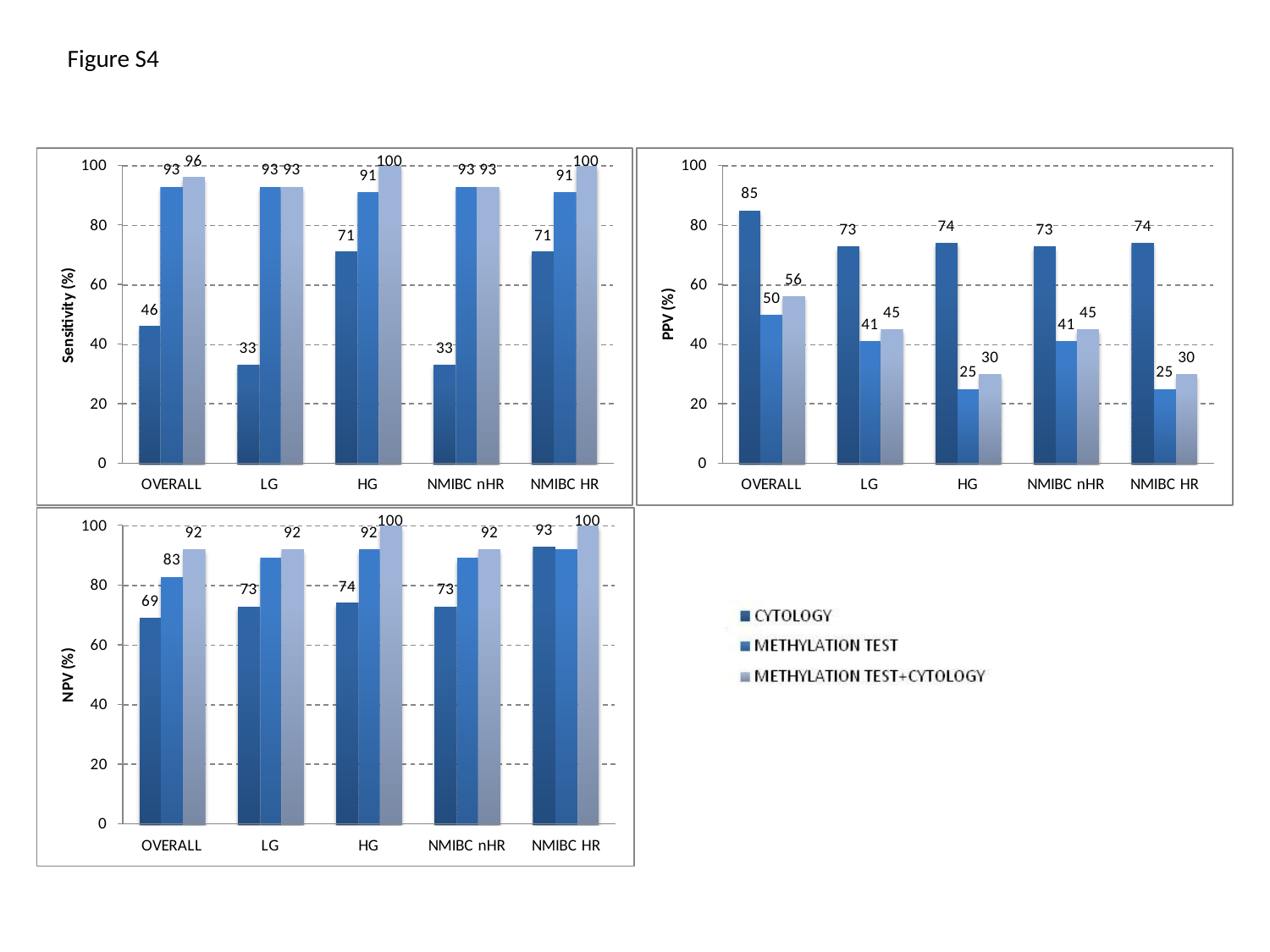

Figure S4

Supplement: Supplementary file 7 — Figure S4. Sensitivity, negative and positive predictive values of urine cytology, the three-gene methylation classifier, and the combined three-gene methylation/cytology classifier in the testing set (N = 308). Overall specificity was 94% for urine cytology, 27% for the three-gene methylation classifier, and 40% for the combined three-gene methylation/cytology classifier. Abbreviations: LG, low-grade; HG, high-grade; NMIBC nHR, non-muscle-invasive bladder cancer non-high risk; NMIBC HR, non-muscle-invasive bladder cancer high risk; NPV, negative predictive value; PPV, positive predictive value. (PPTX 189 kb) [file 13148_2018_496_MOESM7_ESM.pptx]

## Slide 1
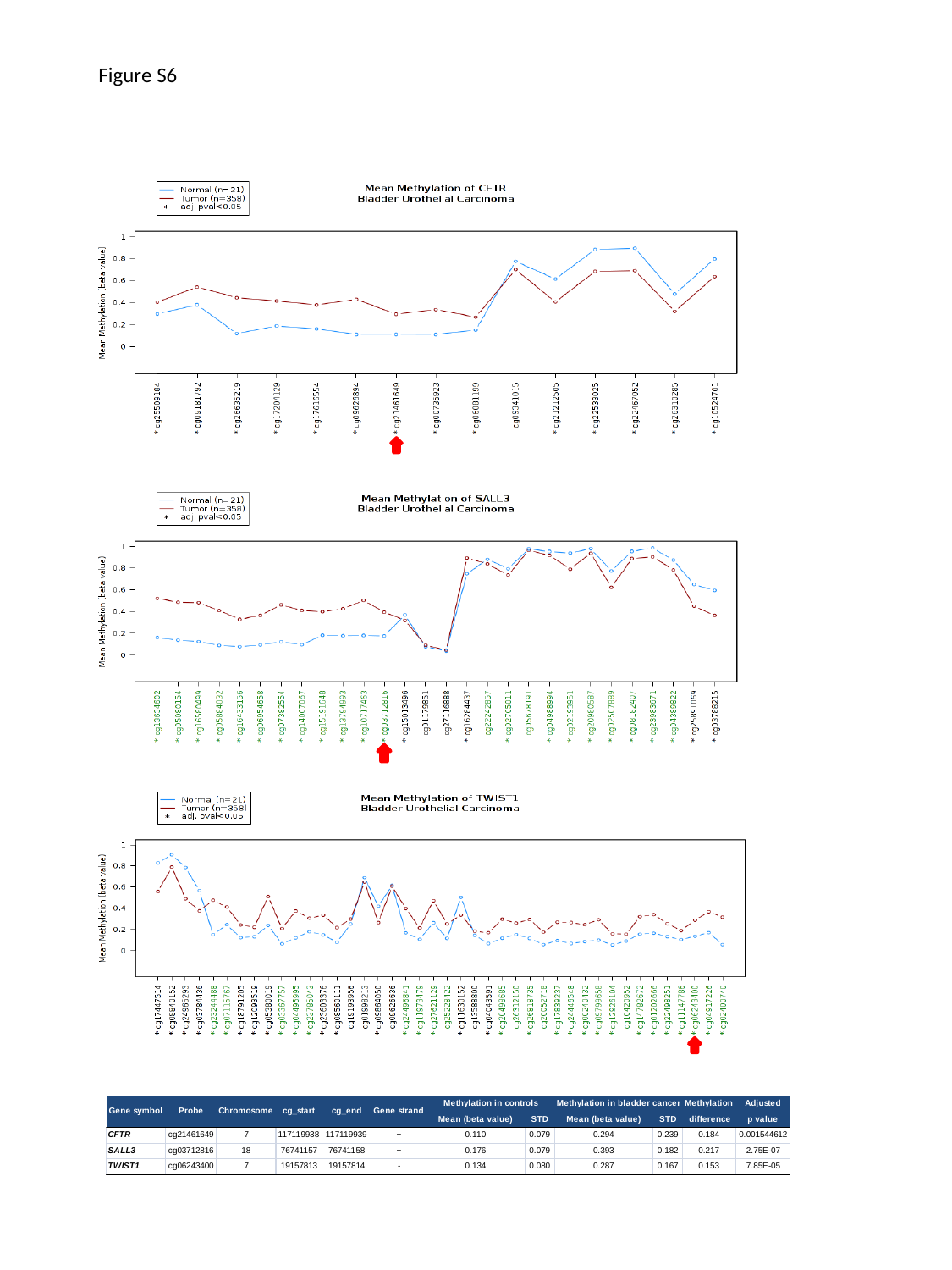

Figure S6

Supplement: Supplementary file 9 — Figure S6. DNA methylation profiles for bladder cancer and control tissue samples for the three-gene classifier. Data obtained from Wanderer Web page: http://maplab.imppc.org/wanderer/. The red arrow indicates the CpG dinucleotide analyzed in each of the three genes. (PPTX 203 kb) [file 13148_2018_496_MOESM9_ESM.pptx]
