# Supplementary material for: Urine cell-based DNA methylation classifier for monitoring bladder cancer
Source: Clin Epigenetics. 2018 May 30;10:71. doi: 10.1186/s13148-018-0496-x (PMC5975622; doi:10.1186/s13148-018-0496-x)
Supplement: Supplementary file 2 — Table S1. Primer sequences used in PCR and pyrosequencing. (DOCX 13 kb) [file 13148_2018_496_MOESM2_ESM.docx]

**Table S1.**Primer sequences used in PCR and pyrosequencing

| **Gene** | **Location** | **Amplicon length** | **Annealing temperature (^o^C)** | **PCR Primer-F** | **PCR Primer-R** | **Sequencing primer** |
| --- | --- | --- | --- | --- | --- | --- |
| ***CDH13*** | 16q23.3 | 350 | 62 | TTTTTTGTTTTAGGTAGGGAAGAG | AAACCAAAATTACCCCACTTAATA | GTGTGTGTGTGTGTGTGTA |
| ***CFTR*** | 7q31.2 | 211 | 58 | GAGGGAGGTTGGGAGTTAGAA | CACACCACCCCTTCCTTTTA | GAGGTTGGGAGTTAGAAT |
| ***NID2*** | 14q21-q22 | 196 | 58 | GGGGATGGGAAGATTTTGATT | ATCTCCTCCAACTCCTAAAACTTC | TTTTGATTTTTTGTTTATTG |
| ***SALL3*** | 18q23 | 276 | 60 | GGGGGTTTGGTTTTGTTTAAT | TCCAACCCTTTACCAATCTCTT | GGTTTTGTAGTTATTT |
| ***TMEFF2*** | 2q32.3 | 101 | 58 | GGGGAGAAGAGAGAAGGAAGGA | ACAACAACAACATCTCCCACA | AAGAGAGAAGGAAGGAGA |
| ***TWIST1*** | 7p21.2 | 167 | 58 | AGGGGAAAGGAGGGTTTAGAA | AAACCCACCCAATAATCAAAT | GAAAGGAGGGTTTAGAAG |
| ***VIM2*** | 10p13 | 290 | 58 | GAGTAGTTTAAGGGTTAAGGTAAG | CTATCTCCCTAACAAACCTCC | TAGTTTAAGGGTTAAGGTA |
