# Supplementary material for: Urine cell-based DNA methylation classifier for monitoring bladder cancer
Source: Clin Epigenetics. 2018 May 30;10:71. doi: 10.1186/s13148-018-0496-x (PMC5975622; doi:10.1186/s13148-018-0496-x)
Supplement: Supplementary file 5 — Table S2. Percentage of methylation for each CpG dinucleotide in the seven selected genes in control and bladder cancer urine samples. Underlined in grey the CpG site used for methylation analysis. Abbreviations: SDV; Standard Deviation. (DOCX 21 kb) [file 13148_2018_496_MOESM5_ESM.docx]

**Table S2.** **Percentage of methylation for each CpG dinucleotide in the seven selected genes in control and bladder cancer urine samples.**

Underlined in grey the CpG site used for methylation analysis.

Abbreviations: SDV; Standard Deviation.
