# Supplementary material for: Urine cell-based DNA methylation classifier for monitoring bladder cancer
Source: Clin Epigenetics. 2018 May 30;10:71. doi: 10.1186/s13148-018-0496-x (PMC5975622; doi:10.1186/s13148-018-0496-x)
Supplement: Supplementary file 6 — Table S3. Diagnostic performance of the three-gene methylation classifier, cytology, and the combined methylation/cytology classifier in the training and testing subset of samples with cytology available. Abbreviations: LG, Low Grade; HG, High Grade; AUC, area under curve; MIBC, muscle invasive bladder cancer; NMIBC, Non-Muscle Invasive Bladder Cancer; NPV, Negative Predictive Value; PPV, Positive Predictive Value; SN, Sensitivity; SP, Specificity; BC, Bladder Cancer; C, Control; R-PFBC, Recurrent Patients in Follow up for Bladder Cancer; NR-PFBC, Non Recurrent Patients in Follow up for Bladder Cancer. (DOCX 20 kb) [file 13148_2018_496_MOESM6_ESM.docx]

**Table S3.** Diagnostic performance of the 3-gene methylation classifier, cytology and the combined methylation/cytology classifier in the training and testing subset of samples with cytology available.

Abbreviations: LG, Low Grade; HG, High Grade; AUC, area under curve; MIBC, muscle invasive bladder cancer; NMIBC, Non-Muscle Invasive Bladder Cancer; NPV, Negative Predictive Value; PPV, Positive Predictive Value; SN, Sensitivity; SP, Specificity; BC, Bladder Cancer; C, Control; R-PFBC, Recurrent Patients in Follow up for Bladder Cancer; NR-PFBC, Non Recurrent Patients in Follow up for Bladder Cancer.
