# Supplementary material for: Urine cell-based DNA methylation classifier for monitoring bladder cancer
Source: Clin Epigenetics. 2018 May 30;10:71. doi: 10.1186/s13148-018-0496-x (PMC5975622; doi:10.1186/s13148-018-0496-x)
Supplement: Supplementary file 8 — Figure S5. Flow diagram of participants in the cross-sectional study according a) to the three-gene methylation classifier and cytology results and b) to the combined three-gene methylation/cytology classifier. Abbreviations: R-PFBC, recurrent patients in follow-up for bladder cancer; NR-PFBC, non-recurrent patients in follow-up for bladder cancer; Cytol, cytology; NA, non-available; Test, combined three-gene methylation/cytology classifier. (PPTX 85 kb) [file 13148_2018_496_MOESM8_ESM.pptx]

## Slide 1
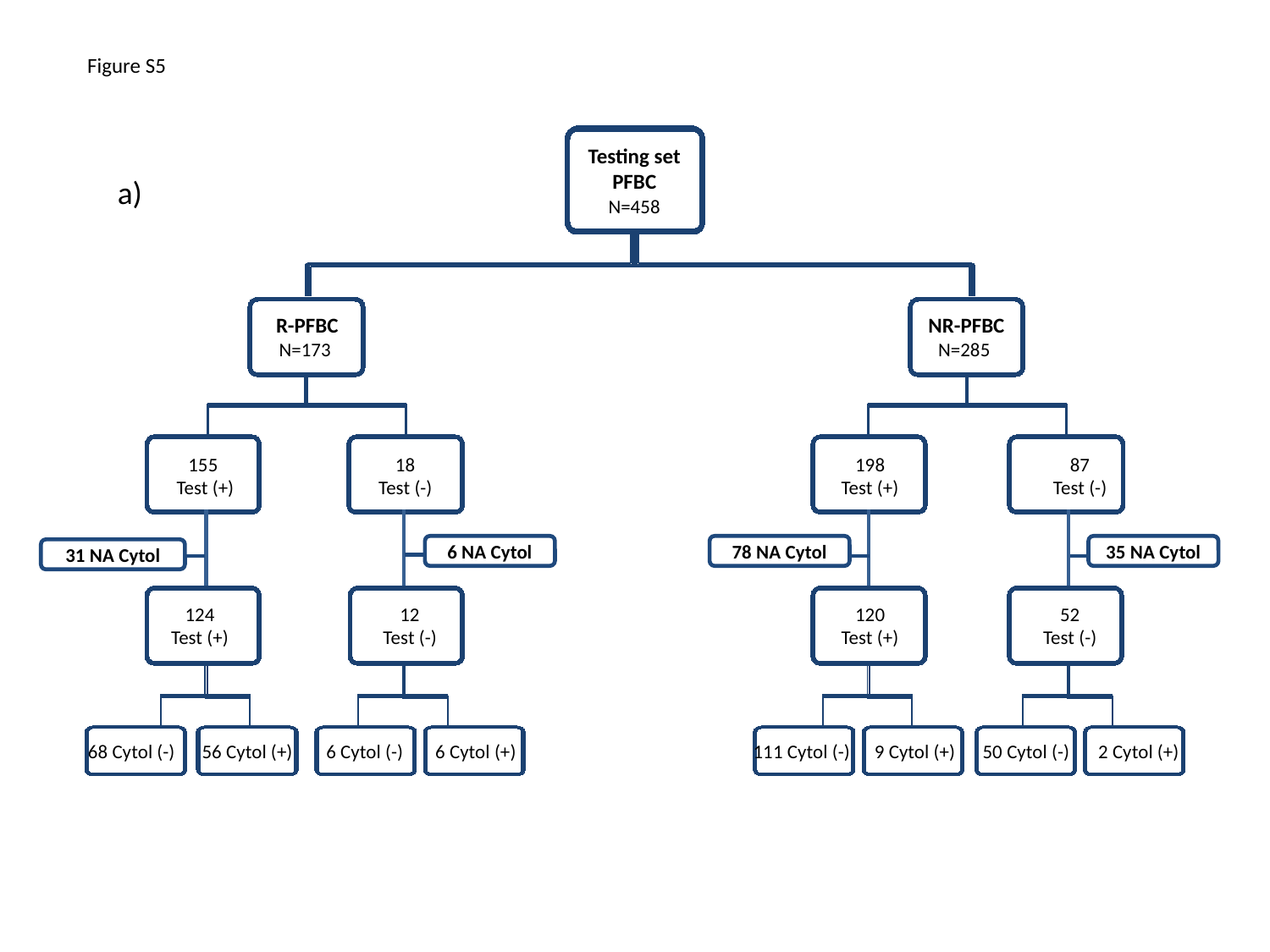

Figure S5
Testing set
PFBC
a)
N=458
R-PFBC
N=173
NR-PFBC
N=285
155
Test (+)
18
Test (-)
198
Test (+)
87
Test (-)
6 NA Cytol
78 NA Cytol
35 NA Cytol
31 NA Cytol
124
Test (+)
12
Test (-)
120
Test (+)
52
Test (-)
68 Cytol (-)
56 Cytol (+)
6 Cytol (-)
6 Cytol (+)
111 Cytol (-)
9 Cytol (+)
50 Cytol (-)
2 Cytol (+)

## Slide 2
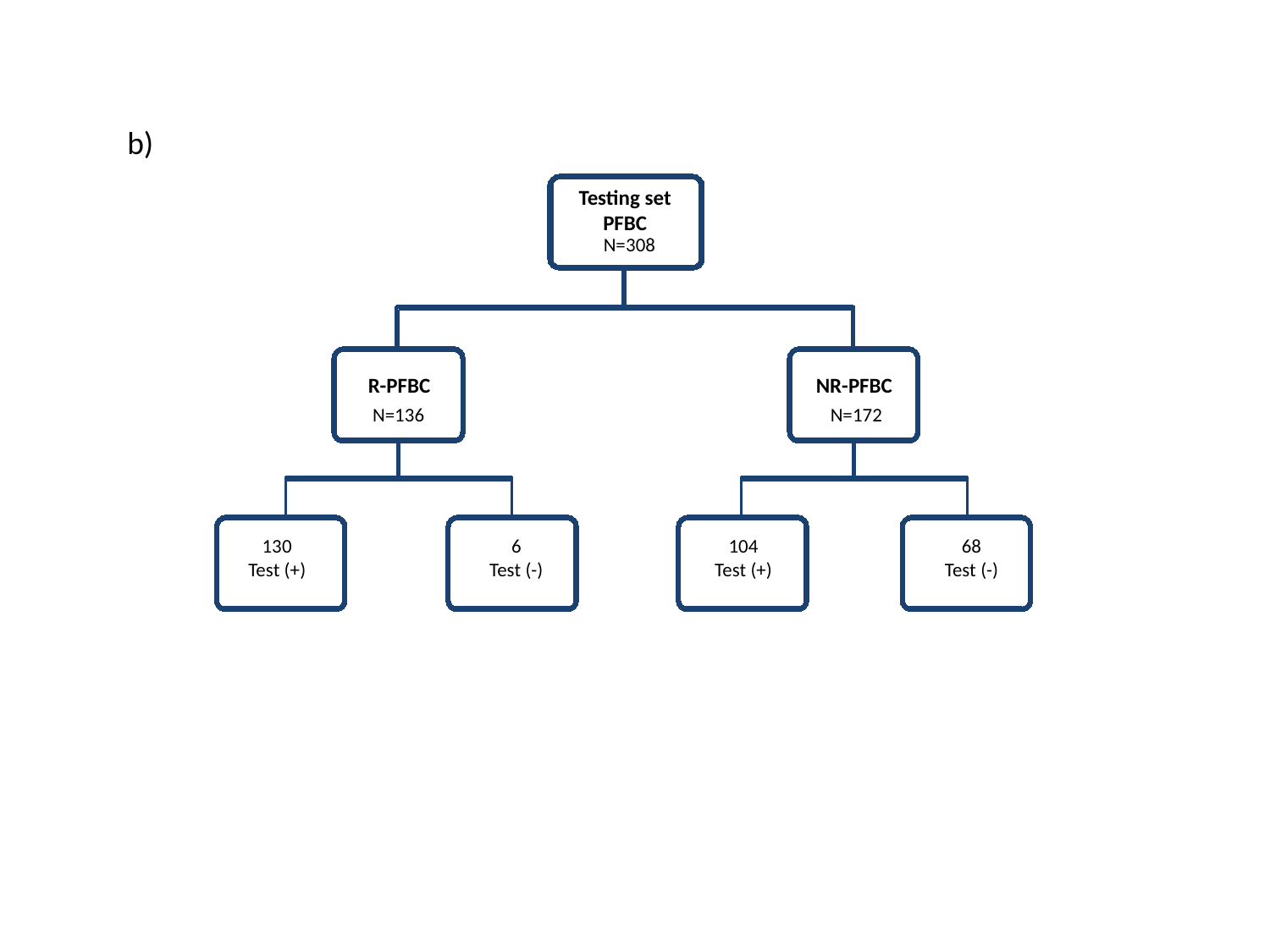

b)
Testing set
PFBC
N=308
R-PFBC
NR-PFBC
N=136
N=172
130
Test (+)
6
Test (-)
104
Test (+)
68
Test (-)
